# Enhanced Phosphorus Removal from Metallurgical Grade Silicon by the Combined Process of Si-Cu Solvent Refining and CaO-CaF_2_-CaCl_2_ Slag Treatment

**DOI:** 10.3390/ma18112502

**Published:** 2025-05-26

**Authors:** Xinlin Wei, Qing Zhao, Juncheng Li, Jingwei Li

**Affiliations:** 1 School of Material Science and Engineering, Jiangsu University, Zhenjiang 212013, China; 2222205134@stmail.ujs.edu.cn; 2 Key Laboratory for Ecological Metallurgy of Multimetallic Mineral (Ministry of Education), Northeastern University, Shenyang 110819, China; zhaoq@smm.neu.edu.cn; 3 College of Energy and Environment Science, Yunnan Normal University, Kunming 650500, China; 4 School of Materials Science and Engineering, Hefei University of Technology, Hefei 230009, China; jwli@hfut.edu.cn

**Keywords:** metallurgical grade silicon, phosphorus removal, slag treatment, solvent refining, microstructure

## Abstract

To develop a high-efficiency process for removing phosphorus (P) from metallurgical grade silicon, a novel method of combining Si-Cu solvent refining and CaO-CaF_2_-CaCl_2_ slag treatment was investigated through simultaneously re-constructing P-containing phases of CaCu_2_Si_2_ in the Si-Cu alloy and Ca_3_P_2_ in the slag. After acid leaching, P-containing phases can be eliminated, whereupon high-purity silicon could be recovered from the Si-Cu alloy. The effect of slag components and alloy composition on the P removal efficiency was studied systematically. When the Si-40 wt.% Cu alloy is treated with 20 wt.% CaO-32 wt.% CaF_2_-48 wt.% CaCl_2_ slag for 60 min at 1400 °C, the P removal efficiency reaches 90.1%. Furthermore, the mechanism of enhanced P removal was also discussed. It was indicated that a silicothermal reduction reaction occurred between CaO and Si, which caused Ca to migrate into the alloy and precipitate the P-containing CaCu_2_Si_2_ in the Si-Cu alloy. Simultaneously, P in silicon is reduced to P^3−^ at the slag–alloy interface, forming Ca_3_P_2_ in the slag, thereby establishing a dual-path purification mechanism. Hence, this study provides new insight into silicon high-efficiency purification from economical and practical considerations.

## 1. Introduction

With the rapid development of the photovoltaic industry in recent years, the demand for solar-grade silicon (SoG-Si) has steadily increased. Metallurgical methods have garnered significant attention due to their low energy consumption, simplified process, and environmentally friendly by-products [1]. Depending on the nature of impurities present in silicon, various refining techniques such as slag treatment [2,3], solvent refining [4,5,6,7,8], acid leaching [9,10], vacuum refining [10,11], electron beam melting [12], and directional solidification [13] are employed to remove different types of impurities. Given the large segregation coefficient (a parameter describing the distribution ratio of impurities between solid silicon crystals and molten silicon during crystal growth) of P at 0.35 [13], its removal during metallurgical purification remains a challenging issue. Solvent refining, characterized by its lower operating temperature, is particularly effective for P removal at low temperatures, offering high impurity removal efficiency and the capability to eliminate multiple impurities simultaneously. This method is considered one of the most promising low-cost metallurgical technologies for large-scale industrial applications and can also achieve significant energy savings. Consequently, the purification of MG-Si via solvent refining has attracted considerable attention from researchers over the past few decades.

Solvent refining leverages the recrystallization behavior of Si within the alloy melt, leading to the redistribution of impurities in Si. Commonly used alloy fluxes include Sn [4,7], Al [5,8], Ca [6], Fe [14], and Cu [14,15]. Copper exhibits a strong affinity with most impurities in Si forming stable intermetallic compounds, such as Al, Ca, and P. It has very low solubility in solid Si, rapid diffusion in molten Si, and a significant density difference between the Si phase and the Si-Cu phase facilitating separation through heavy medium techniques. As a result, the Si-Cu system is one of the most extensively studied [14]. Mitrašinović et al. utilized Si-Cu alloys to remove impurities from Si, achieving an impurity content in the residual Si-Cu alloy ten times higher than that of pure Si. However, the removal efficiency for non-metallic impurities like P was relatively low [15]. Zhang et al. introduced Ti and Ca into the Si-Cu alloy, resulting in the formation of TiSi_2_ and CaCu_2_Si_2_ phases with high P concentrations in the Cu_3_Si alloy phase, demonstrating that adding Ti and Ca can effectively enrich P in the Si-Cu alloy [16]. Huang et al. proposed a method combining Si-Cu solvent refining and acid leaching to remove P from Si. The experimental results showed that the removal rate of P from MG-Si increased from 23.2% to 42.2% with the increase in Cu content of Si-Cu alloy [17]. Furthermore, with the addition of a small amount of Ca to the Si-Cu alloy, the P-containing CaCu_2_Si_2_ phase was observed in the Si-Cu alloy and the P removal efficiency increased from 27% to 82% when adding 5 wt.% Ca [18]. Due to the difficulty in obtaining elemental Ca, some scholars noted that CaO-rich refining slags were more likely to generate Ca in Si during slag treatment, effectively promoting Ca incorporation into Si [19,20]. These findings have inspired many researchers to explore introducing Ca into Si via slag treatment technology to enhance P removal. Kawamura et al. performed slag treatment on MG-Si using CaO-CaF_2_, finding that the refining slag contained a certain amount of SiO_2_, and Si contained 1.3–6.6 wt.% Ca. The acid leaching of silicon after the reaction with slag was conducted with aqua regia. The removal fraction of P was in the range of 58–92%; this process can effectively achieve the removal of P impurity [20]. Cheng et al. used CaO-SiO_2_-CaF_2_ flux to refine MG-Si, significantly increasing the Ca content in Si and detecting a large amount of CaSi_2_ phase via EDS analysis [2]. Shin et al. used CaO-CaF_2_ to dephosphorization and refine the Si-Mn alloy, where CaO in the slag reacted with Si in the Si-Mn alloy, successfully reducing CaO in the CaO-CaF_2_ slag to Ca and facilitating Ca migration from the slag to the metal phase, greatly enhancing P removal from the Si-Mn melt [21]. These experiments confirmed that CaO-rich slags readily undergo silicothermal reactions with Si. Therefore, it is feasible to introduce Ca into Si through CaO slag. On the one hand, the addition of CaO reduces the cost compared with Ca. On the other hand, under conditions of low oxygen partial pressure and high basicity slag, a part of P in Si will be reduced to P^3−^ and enter the slag phase, thereby establishing a foundation for efficient P removal.

Table 1 highlights the key findings and limitations of previous P removal methods regarding Si-Cu solvent refining and CaO-based slag treatment. To effectively eliminate P impurities from silicon, this study adopts a combined purification process of Si-Cu solvent refining with CaO-CaF_2_-CaCl_2_ slag treatment. At relatively low temperatures, this method concurrently reconstructs the P-containing phase CaCu_2_Si_2_ in the alloy and the P-containing compound Ca_3_P_2_ in the slag; the dual removal path of P has been achieved. Additionally, the effects of slag composition and Cu content on P removal were investigated, and the corresponding separation and purification mechanisms were also discussed.

## 2. Materials and Experimental

In this experiment, P was doped into polycrystalline Si to increase the P content in Si, to facilitate the observation and analysis of the distribution of P in Si. Thus, Cu-Si-1 wt.% P alloy doped with P was prepared using SoG-Si (6N), Cu (4N), and Si-6 wt.%P master alloy for all experiments. The refining slags were composed of analytical grade CaO, CaF_2_, and CaCl_2_. The reagents used in this study were provided by Sinopharm Chemical Reagent Co., Ltd. (Shanghai, China).

10 g P-doped Si-Cu alloy and 10 g of uniformly mixed CaO-CaF_2_-CaCl_2_ refining slag are placed in a high-purity graphite crucible. Their compositions are shown in Table 2. The samples were placed in a VTL-1700 tube furnace for melting. The mixture was heated at a rate of 10 °C/min to 1400 °C in an argon atmosphere and held for 1 h to homogenize the melt. Then, the samples were cooled at a rate of 5 °C/min to room temperature.

The mineral component of the refined slags was analyzed by X-ray diffraction (XRD; TTRIII, Tokyo, Japan), and the refined alloys were characterized by an X-ray fluorescence spectrometer (XRF; ARL Advant’X IntelliPower-4200, Massachusetts, MA, USA). The microstructure and elemental distribution of the refined alloy were characterized by scanning electron microscopy and energy dispersive spectroscopy (FEI Quanta250, Hillsboro, OR, USA). The refined alloy was placed in an agate mortar and ground into fine particles. Then, the ground sample was sieved through a stainless steel standard sieve to collect alloy particles with a particle size of less than 150 µm; 2.0 g of alloy powder was weighed and poured into a Teflon beaker, which was placed on a constant temperature magnetic stirrer set at 343 K temperature and a stirring speed of 400 rpm. First, the powder was leached with 20 mL of 2 mol/L aqua regia for 5 h, and then treated with 20 mL of 1 mol/L HNO_3_ + trace HF for 2 h. Finally, 20 mL of 1 mol/L HNO_3_ was employed to remove minor impurities that had been reversely adsorbed onto the surface of the silicon particles, thereby obtaining pure silicon. At the end of each stage, the remaining solid was washed, after acid washing multiple times with deionized water until the pH value of the solution was 7, and then the sample was placed in a vacuum drying oven for drying. After the acid leaching, the P content in the primary silicon was determined by an inductively coupled plasma atomic emission spectrometer (ICP-AES; VISTA-MPX, Santa Clara, CA, USA), and the P removal rate was calculated accordingly.

## 3. Results and Discussion

### 3.1. Morphology Characteristics of Si-Cu Alloy Treated by CaO-CaF_2_-CaCl_2_ Slag

**Figure 1:**
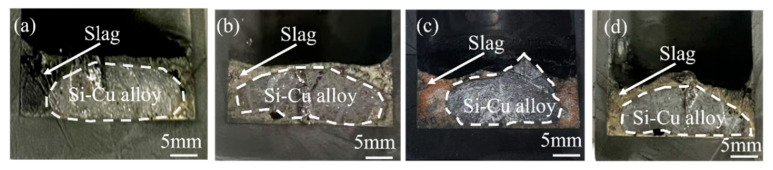
Cross-sections of refined samples: (**a**) E2; (**b**) E4; (**c**) E7; (**d**) E8.

Figure 1 shows the cross-sections of samples after the combined process of Si-Cu solvent refining and CaO-CaF_2_-CaCl_2_ slag treatment. As can be seen in Figure 1, the boundary between slag and alloy was obvious, and Si-Cu alloy was completely wrapped by refined slag. This phenomenon can be attributed to the high proportion of CaCl_2_ and CaF_2_ in the refined slag, resulting in low viscosity and excellent fluidity. Consequently, the refined slag maintained full contact with the silicon melt. This also means that sufficient interaction area between the slag and the alloy promoted the slag–alloy reaction, thereby enhancing the P removal efficiency from MG-Si.

**Figure 2:**
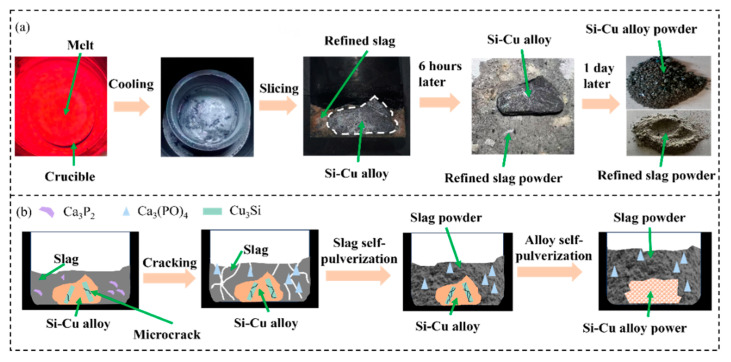
(**a**) Separation of Si-50 wt.% Cu alloy from 20 wt.% CaO-48 wt.% CaCl_2_-32 wt.% CaF_2_ slag; (**b**) schematic diagram of self-pulverization of slag and alloy.

Figure 2a shows the separation of Si-50 wt.% Cu alloy from 20 wt.% CaO-48 wt.% CaCl_2_-32 wt.% CaF_2_ slag. When the refined sample was taken out of the crucible, it was found that the slag adhered to the surface of the alloy. However, the refined slag self-pulverized after being placed at room temperature for 6 h, which may be attributed to the exothermic reaction of Ca_3_P_2_ in the refined slag upon contact with oxygen in the air, leading to the formation of Ca_3_(PO_4_)_2_ [23]. During this process, the heat released from the reaction caused volumetric expansion of the slag. The resultant thermal expansion induced significant internal stress within the refined slag, which led to the self-pulverization of the slag into fine particles. This facilitates the separation of alloy from the refined slag, avoiding the loss of large amounts of primary silicon. Furthermore, when Si-Cu alloy was placed at room temperature for one day, it was easy to crush to powder, conducing to the subsequent pickling process. It could be ascribed to the accumulation of internal stress caused by the volume difference of precipitated phases of Si and Cu_3_Si in the Si-Cu alloy, resulting in a natural tendency of powder [24]. The schematic diagram of the self-pulverization of slag and alloy is shown in Figure 2b. As can be seen, the phase transformation from Ca_3_P_2_ to Ca_3_(PO_4_)_2_ caused slag self-pulverization, while the development of microcrack between precipitated phases of Si and Cu_3_Si induced alloy self-pulverization.

**Figure 3:**
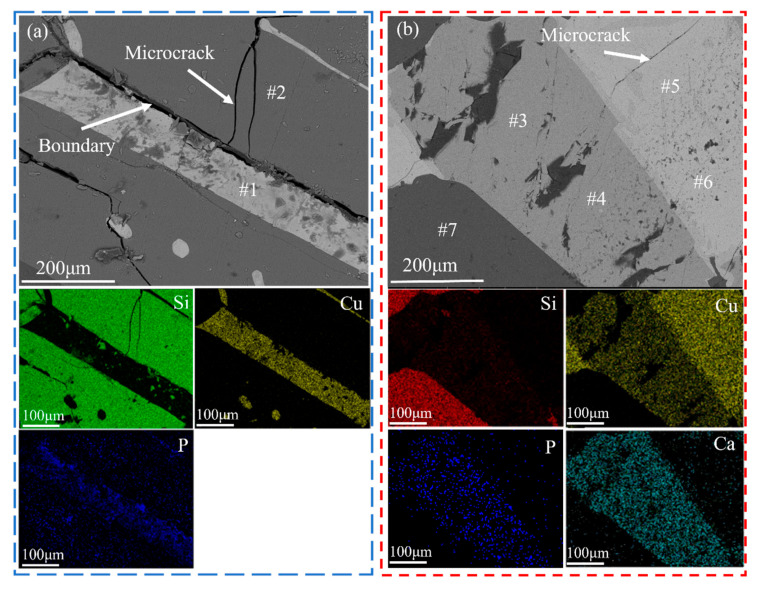
Microstructure and mapping analysis of refined Si-Cu alloys: (**a**) E0; (**b**) E7.

Figure 3 shows the microstructure and mapping analysis of refined Si-Cu alloys without (Figure 3a) and with (Figure 3b) slag treatment. As shown in Figure 3a, the refined Si-Cu alloy consists of a dark gray Si phase and a grayish-white Si-Cu phase. With the help of EDS mapping analysis, P was concentrated in the Si-Cu phase, rather than the Si phase. However, the new light gray rod-like phase that was rich in P appeared in the Si-Cu alloy in Figure 3b. Table 3 summarizes the EDS point analysis results and the new phase could be CaCu_2_Si_2_ (#3, #4). Notably, the P content in the CaCu_2_Si_2_ phase was significantly higher than in the Si-Cu alloy phase (#2, #5, #6), indicating a much higher affinity for P in the CaCu_2_Si_2_ phase in comparison with the Si-Cu phase. This finding is consistent with the experimental results of P segregation in the CaCu_2_Si_2_ alloy phase observed by Huang et al. in the Si-Cu-Ca alloy system [18]. Subsequent acid leaching of the alloy can effectively remove P impurities.

### 3.2. Removal of P Impurity

#### 3.2.1. Effect of CaO Content in Slag

**Figure 4:**
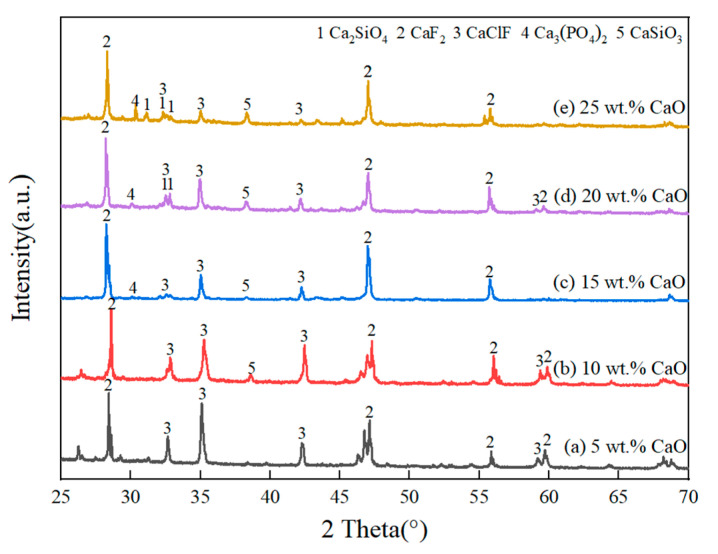
XRD pattern of the refined slags with different CaO contents: (**a**) 5 wt.%, (**b**) 10 wt.%; (**c**) 15 wt.%; (**d**) 20 wt.%; (**e**) 25 wt.%.

Figure 4 shows the XRD pattern of the refined slags with different CaO contents. As the CaO content in the slags increased from 5 wt.% to 25 wt.%, the peak intensity of Ca_2_SiO_4_ and CaSiO_3_ gradually increased. This is because a relatively high CaO content facilitates the formation of SiO_2_ via the silicothermal reaction between CaO in slag and Si in alloy. The pre-generated SiO_2_ further reacted with excessive CaO to form Ca_2_SiO_4_ and CaSiO_3_. Table 4 shows the XRF results of refined alloys with different CaO contents, and the linear increase between Ca content in the alloy and CaO content in the slag further confirms the migration of Ca from the slag to the alloy. It is consistent with the fact that the Ca content in silicon increased significantly after using CaO-based slag to purify MG-Si [25]. When the CaO content exceeded 15 wt.%, Ca_3_(PO_4_)_2_ was detected in the slags. Similar results were found in the investigation of removing P from MG-Si using a combined process of Si-Al-Ca solvent refining and CaO-CaF_2_ slag treatment [23]. The reasons could be attributed to P being reduced by CaO to form P^3−^ and subsequently entering the refined slag to form the Ca_3_P_2_ phase during the refining process. After the refining, the slag was exposed to the air and Ca_3_P_2_ was oxidized to form calcium phosphate Ca_3_(PO_4_)_2_.

**Figure 5:**
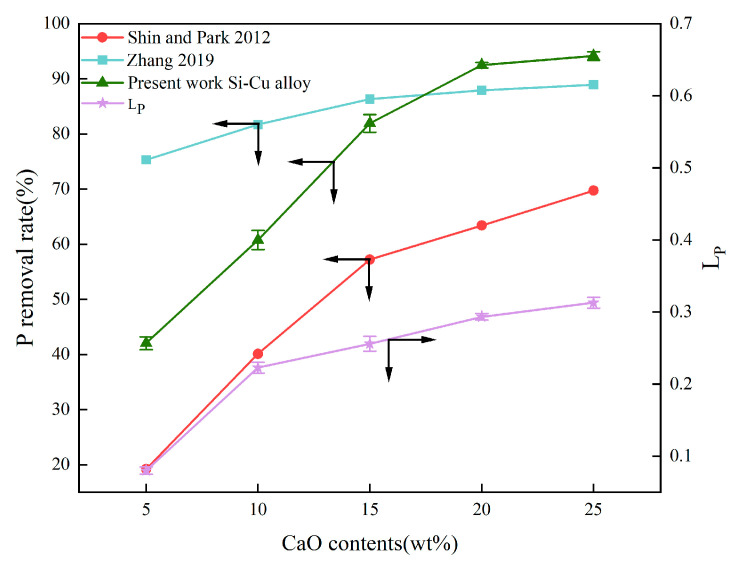
Effect of CaO content on the removal rate and distribution ratio of P [21,23]. The black arrows represent the corresponding coordinate axes.

The effect of CaO content on the removal rate and distribution ratio of P is shown in Figure 5. In the present work, a significant increase in P removal efficiency appeared with an increase in CaO content, up to about 20 wt.%. The removal rate of P increases linearly with the content of CaO in the slag was also reported by other researchers as shown in Figure 5 [21,23]. This is because, on the one hand, higher CaO content ensures sufficient CaO to react with Si so that a large amount of Ca migrates into the alloy, which can effectively reduce the segregation coefficient of P between solid Si and Si-Cu melt; on the other hand, high CaO content means high O^2−^ concentration, facilitating the reduction of P to P^3−^ via the following Reaction (1):

[P] + 3/2O^2−^ = P^3−^ + 3/4O_2_ (1)

It can be seen in Figure 5 that the removal rate of P has a negligible increase when CaO content is more than 20 wt.%, which could be attributed to CaO becoming saturated with the CaO addition of more than 20 wt.%. In addition, excessive CaO addition would increase the slag melting point and reduce the liquid slag phase, unfavorable for the reaction between CaO and Si. It was also found in Figure 5 that the CaO content in the refined slags significantly influenced the distribution coefficient of P (L_P_), which can be expressed by Equation (2) as follows:

(2) $$L_{P}=\frac{\left(M\right)}{\left[M\right]}$$

where (M) and [M] refer to the concentration of impurity P in the slag and in the metal after refining, respectively. As the CaO content in the slag increased from 5 wt.% to 25 wt.%, L_P_ increased from 0.08 to 0.31. This is due to the increase in the oxygen potential in the slag caused by the increasing CaO content, resulting in the possibility of P reduction and entering the slag phase in the form of Ca_3_P_2_ [26].

#### 3.2.2. Effect of Fluorine-to-Chlorine Ratios in Slag

**Figure 6:**
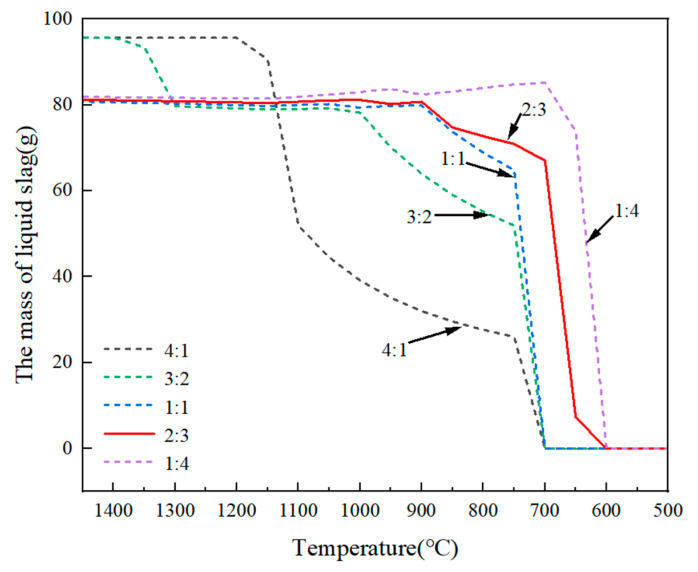
Effect of mass ratios of fluorine-to-chlorine on the content of liquid slag and solidification temperature.

Figure 6 shows the amount of liquid slag and its solidification temperature determined by the equilibrium computation of FactSage 8.3 with the application of the databases of FactPS, Ftoxid, Ftsalt, and FSupsi. As the increasing mass ratio of fluorine-to-chlorine (the mass ratio of CaF_2_ to CaCl_2_ in the slag) from 1:4 to 4:1, the solidification temperature of refining slag increased from 600 °C to 700 °C. The larger amount of liquid slag and lower solidification temperature can effectively reduce the mass transfer resistance of the slag–alloy interface, increase the interaction area between the two phases, accelerate the migration of P from the alloy to the slag phase, and thus significantly improve the P removal efficiency. Therefore, appropriately increasing the proportion of CaCl_2_ in the slag benefited P removal.

**Figure 7:**
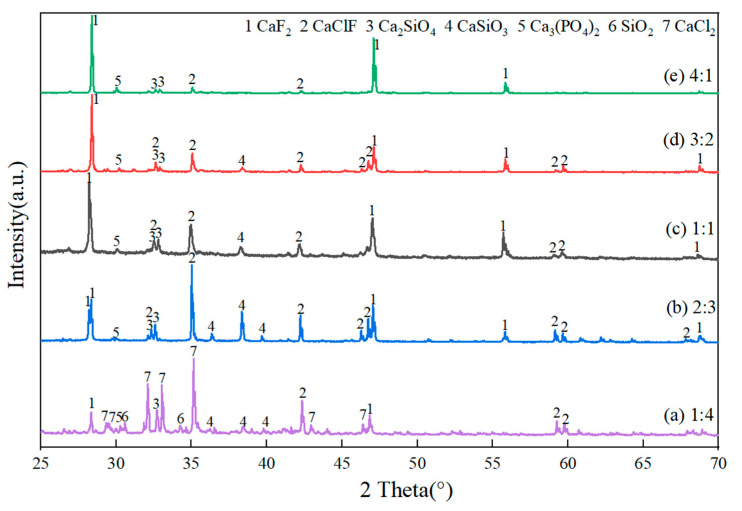
XRD pattern of the refined slags with different mass ratios of fluorine to chlorine: (**a**) E9; (**b**) E8; (**c**) E4; (**d**) E7; (**e**) E6.

Figure 7 shows the XRD pattern of the refined slags with different mass ratios of fluorine-to-chlorine. In Figure 7, the main phases in the refined slag were CaF_2_ and CaClF, whose peak intensity increased with the mass ratio of fluorine-to-chlorine from 1:4 to 4:1. The peak intensity of Ca_3_(PO_4_)_2_ remained unchanged with the mass ratios of fluorine to chlorine increasing from 1:4 to 4:1. Notably, the highest peak intensity of CaSiO_3_ appeared with the mass ratios of fluorine-to-chlorine being 2:3. This may be because, on the one hand, the CaF_2_ component in the refining slag provides F^−^ ions and breaks the silicate network structure, thus reducing the viscosity of the slag system and promoting the silicothermic reduction reaction [27,28]; on the other hand, CaCl_2_ and SiO_2_ reacted at a high temperature [22], ensuring the alkalinity balance of the slag system. Therefore, more CaO can participate in the reduction reaction of silicon and P at the slag–alloy interface, enhancing the removal of P from MG-Si.

**Figure 8:**
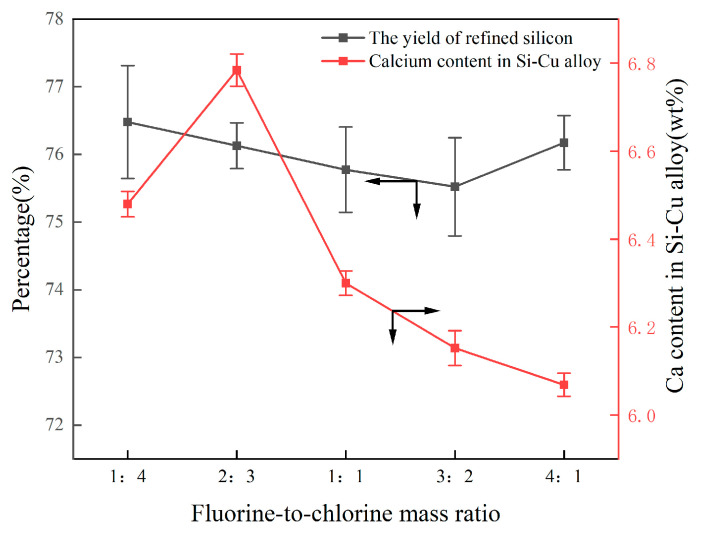
The effect of mass ratios of fluorine-to-chlorine on the Ca content in the alloy and the yield of refined silicon. The black arrows represent the corresponding coordinate axes.

The effect of different mass ratios of fluorine-to-chlorine on the Ca content in the alloy and the yield of refined silicon is shown in Figure 8. Obviously, the mass ratios of fluorine-to-chlorine had a negligible effect on Ca migration and the yield of refined Si.

**Figure 9:**
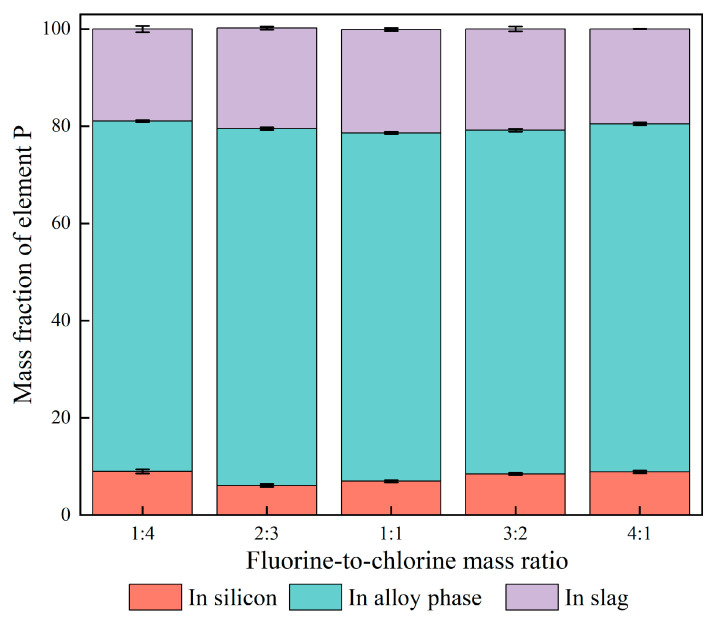
Distribution of P in different phases with fluorine-to-chlorine ratios.

Figure 9 shows the distribution of P in different phases with varying fluorine-to-chlorine ratios. With the mass ratio of fluorine to chlorine from 1:4 to 4:1, the P content in the slag system fluctuates around 20 wt.%. When the mass ratio of fluorine-to-chlorine in the slag was 2:3, 73.23% P was concentrated in the alloy phase (Cu_3_Si, CaCu_2_Si_2_), with its corresponding removal rate reaching 93.7%. As a result, the optimum composition of the ternary slag system used in this study was 20 wt.% CaO-32 wt.% CaF_2_-48 wt.% CaCl_2_.

#### 3.2.3. Effect of Alloy Composition

**Figure 10:**
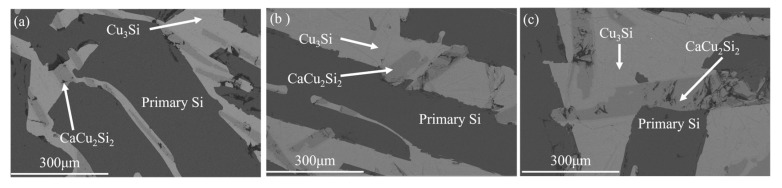
Morphology of refined alloys with different Cu content (**a**) 30 wt.% Cu; (**b**) 40 wt.% Cu; (**c**) 50 wt.% Cu.

Figure 10 shows the microstructure of refined alloys with different Cu contents. As illustrated in Figure 10, with the increase in Cu content in the alloy, the proportion of the Cu_3_Si phase in the refined alloys increased. This is because the increase in Cu content in the alloy promotes the formation of the alloy phase and helps to dissolve P impurity in Si [29]. In addition, the CaCu_2_Si_2_ phase exhibited a gradual increase in thickness with the increase in Cu content in the alloy.

**Figure 11:**
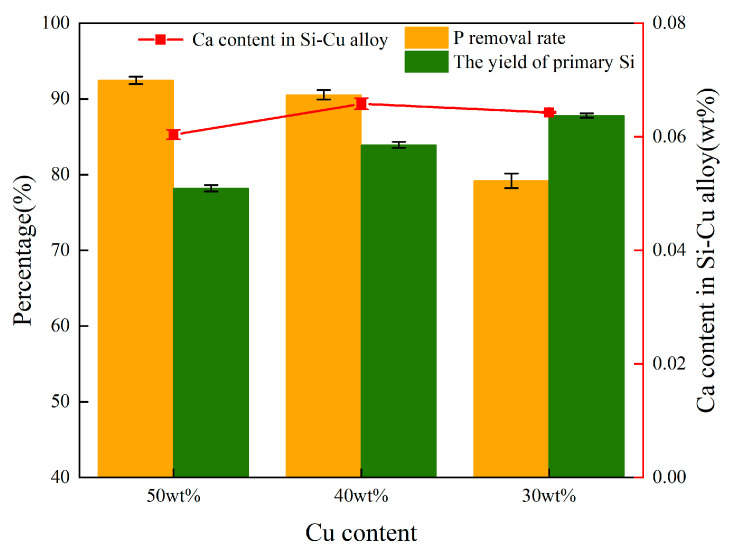
Relationship of alloy composition to Ca content, P removal efficiency and the silicon yield in refined Si-Cu alloy.

The Ca content in the alloy, the removal rate of P, and the recovery rate of primary silicon are plotted in Figure 11. It can be seen that when the Cu content increases from 30 wt.% to 50 wt.%, the fluctuation range of Ca content in the alloy is less than 0.4 wt.%, indicating that Cu has no significant regulatory effect on the migration behavior of Ca. However, the formation of the CaCu_2_Si_2_ phase is closely related to the Ca content. Therefore, it is believed that the change in Cu content has a relatively small influence on the formation of the CaCu_2_Si_2_ phase. However, the increase in Cu content significantly enhanced the removal efficiency of P from 78.5% (30 wt.% Cu) to 93.7% (50 wt.% Cu). This was because the addition of Cu to Si melt not only led to the thermodynamic instability of P [30]. During the solidification process, the segregation coefficient of P decreases with the increase in Cu content, promoting P to be more inclined to enrich in the Cu-rich liquid region. Combined with the EDS surface scanning results in Figure 3b, the P atoms enriched in the liquid phase show a significant affinity for the CaCu_2_Si_2_ phase, thereby effectively enhancing the probability of P and Ca atoms combining at the solid-liquid interface to form the CaCu_2_Si_2_ phase. Furthermore, the increase in Cu content reduces the Si output in the alloy. This is because during the refining process, a small amount of Si is oxidized to SiO_2_, and the Si-Cu alloy phase consumes some Si. Considering the cost of solvent refining and the yield of refined Si, Si-40 wt.% Cu alloy was chosen as the optimum alloy composition in this study.

### 3.3. Mechanism of P Removal

Based on Figure 4 and Figure 7, it is evident that P exists in the refining slag as calcium phosphate Ca_3_(PO_4_)_2_, rather than Ca_3_P_2_. The reason may be that Si can form Ca_3_P_2_ during the combined process of Si-Cu solvent refining and CaO-CaF_2_-CaCl_2_ slag treatment via Equation (3) [25]:

4P + 6CaO + 3Si = 3SiO_2_ + 2Ca_3_P_2 _ (3)

However, Ca_3_P_2_ had extremely strong activity and easily reacted with water vapor and oxygen when exposed to air [23]. Therefore, the generated Ca_3_P_2_ will react with O_2_ and steam, and the reaction equation is as follows:

Ca_3_P_2_ + 4O_2_ = Ca_3_(PO_4_)_2_ (4)

Ca_3_P_2_ + 3H_2_O = 3CaO + 2PH_3_ (5)

Ca_3_P_2_ + 6H_2_O = 3Ca(OH)_2_ + 2PH_3_ (6)

According to Figure 9, the P content in the alloy is much higher than that in the slag phase, indicating that P is more likely to be enriched in the alloy phase. Table 5 shows the studies of the occurrence state of P in alloys by relevant researchers [5,18,31,32]. It can be seen from Table 5 that P usually existed in the form of solute atoms in the alloy phase. In particular, to confirm the state of the P-containing phase in Si-Cu-Ca, Huang et al. used HCl to dissolve the phase in different alloys based on its acid sensitivity. The atomic percentage of P in Si-Cu-Ca residue did not change significantly after acid leaching. It can be inferred that when the Si-Cu-Ca melt solidified, P was uniformly dissolved in the CaCu_2_Si_2_ phase instead of forming Ca_3_P_2_.

**Figure 12:**
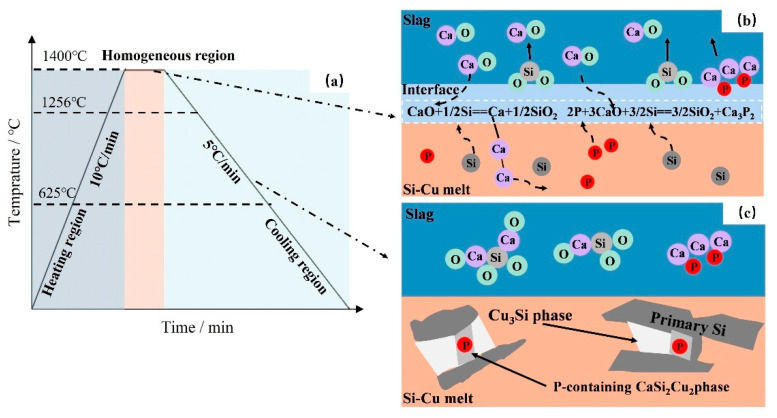
(**a**) Schematic diagram of suitable heat treatment condition; (**b**) schematic diagram of the interfacial reaction between Si-Cu melt and slag; (**c**) phase precipitation during the solidification process.

To analyze the interaction between slag and alloy phases more intuitively, a P removal reaction model between CaO-CaF_2_-CaCl_2_ slag and Si Cu alloy was established, as shown in Figure 12. The EDS analysis results in Table 3 of Section 3.1 and the presence of P-containing substances in the refining slag in Figure 6 confirm that approximately 20% of the P in the alloy migrates to the slag phase (in the form of Ca_3_P_2_), while the remaining P in the melt exists in the form of solid solution in the CaCu_2_Si_2_ phase. The removal efficiency of P is positively correlated with the CaO content in the slag system. Under the high-temperature refining condition of 1400 °C, both the alloy and the slag are in the liquid state, and Reactions (3) and (7) occur at the interface between slag and alloy:

CaO + 1/2Si = Ca + 1/2SiO_2_ (7)

With the progress of the reaction, the Ca_3_P_2_ and SiO_2_ generated on the slag–alloy interface gradually migrate to the slag phase. It is worth emphasizing that the synergistic effect of CaCl_2_ and CaF_2_ in the slag phase significantly enhances the fluidity of the slag phase and accelerates the interfacial mass transfer process. Meanwhile, the generated calcium continuously diffuses from the slag–alloy interface into the alloy phase, resulting in a continuous increase in the calcium content in the alloy. During this process, the Ca content in the alloy depends on the CaO content in the slag. During the solidification process, the preferred precipitation of the primary crystalline silicon phase triggers the solute redistribution effect and the P concentration gradient at the solidification front forces the solute to migrate toward the solid-liquid interface. At this point, the supersaturated Ca, Cu, and P in the alloy start to form the P-containing CaCu_2_Si_2_ phase at the front of the primary Si solidification. As the melt temperature further decreases, P continuously diffuses from silicon to the solid-liquid interface, driving the P-containing phase to grow. Especially in the later stage of solidification, the large-scale precipitation of the CaCu_2_Si_2_ phase effectively reduces the concentrations of P and Ca in the melt, and gradually increases with the decrease in melt temperature. With the formation of a large amount of P-containing CaCu_2_Si_2_ phase, the P content in silicon significantly decreases, resulting in a significant reduction in the P impurity content in the primary silicon. This dual-path purification mechanism of slag and alloy provides theoretical support for achieving efficient P removal.

According to the data in Table 1, the existing refining process has technical bottlenecks such as a P removal rate of only 70–82% and excessive energy consumption. To address this challenge, in this study, by coupling the low-melting-point CaF_2_-CaCl_2_ slag agent with the Si-Cu alloy, a dual-path synergistic purification system was innovatively constructed: at the slag-alloy interface, on the one hand, Ca_3_P_2_ was generated through a reduction reaction and diffused into the slag phase to achieve the primary removal of P; on the other hand, by taking advantage of the strong affinity of CaCu_2_Si_2_ relative to P, the residual P impurities are selectively enriched in the alloy phase, and the directional separation of the alloy phase constitutes a key path for efficient P removal. Compared with the traditional single-phase capture mechanism, the synergistic effect of this slag phase reduction P removal and alloy phase enrichment P removal not only significantly reduces the refining energy consumption, but also breaks through the efficiency bottleneck of the existing process through the directional enrichment and deep separation of P by the latter, thereby meeting the urgent demand of the SoG-Si industry for low-energy consumption and high-efficiency production processes.

### 3.4. Recovery of Refined Slag and Cu

In view of the fact that the refining slag contains various calcium-based compounds (CaClF, CaF_2_, CaSiO_3_, Ca_2_SiO_4_ and Ca_3_(PO_4_)_2_), staged treatment is adopted to achieve resource utilization: Firstly, the fluorine-containing components are preliminarily enriched through crushing and density separation. Subsequently, CaCl_2_ in CaClF was extracted by water leaching. After purification, it was used as a deicing agent or desiccant. The fluoride ions in the wastewater were fixed by lime precipitation. The residue is leached step by step with dilute hydrochloric acid–calcium phosphate which is preferentially dissolved and transformed into raw material for phosphate fertilizer, and silicate forms silica gel precipitate to achieve the separation of P and silicon. Silica gel is subjected to high-temperature alkaline melting to form sodium silicate (raw material for building materials) and finally purified to obtain high-grade CaF_2_ (raw material for metallurgy/fluorine chemical industry). The remaining silicate is compounded with cement to form environmentally friendly building materials, and the trace residue is safely landfilled after solidification. As for the Cu in the acid solution, it can be recycled and reused through anode electrolysis in the future. Future studies aim to explore the addition of cryolite (Na_3_AlF_6_) as a flux to improve energy efficiency in the high-temperature treatment process by lowering the melting point of the slag system.

## 4. Conclusions

This paper investigated an innovative route for MG-Si purification by the combined process of Si-Cu solvent refining and CaO-CaF_2_-CaCl_2_ slag treatment. The conclusions can be summarized as follows:

1. In the refining process, impurity P was found to simultaneously concentrate in the P-rich phases of Ca_3_P_2_ in the slag and CaCu_2_Si_2_ in the alloy. The Ca_3_P_2_ tends to be oxidized to calcium phosphate Ca_3_(PO_4_)_2_ when exposed to air, while CaCu_2_Si_2_ could be removed by selective acid leaching.
2. The P removal rate and L_P_ were linearly increased to the content of CaO; the increase in the mass ratios of fluorine-to-chlorine in the slag system had a negligible effect on Ca migration and the yield of refined Si; the removal efficiency of P in the alloy significantly increased as the Cu content increased from 30 wt.% to 50 wt.%, but the yield of Si decreased from 87.59% to 77.89%.
3. The reaction model of CaO-CaF_2_-CaCl_2_ slag and Si-Cu alloy for P removal was established. During the refining process, a portion of the P in the silicon was reduced to P^3−^ at the slag–metal interface and then entered the slag phase in the form of phosphide Ca_3_P_2_. At the same time, a silicothermal reduction reaction occurred between CaO and Si, resulting in the migration of Ca into the alloy and the precipitation of P-rich CaCu_2_Si_2_ in the Si-Cu alloy. When the Si-40 wt.% Cu alloy was treated with 20 wt.% CaO-32 wt.% CaF_2_-48 wt.% CaCl_2_ slag for 60 min at 1400 °C, the P removal rate of refined silicon reached 90.1%.

## Figures and Tables

**Table 1:** Key findings and limitations of previous P removal methods regarding Si-Cu solvent refining and CaO-based slag treatment.

Method	Key Findings	Limitations	References
Si-Cu Alloy	Impurity P concentrated in Cu_3_Si phase	Relatively low removal rate of impurity P (23–42%)	[15,17]
Si-Cu-Ca Alloy	Impurity P concentrated in CaCu_2_Si_2_ phase with the P removal rate up to 82%	Difficulty in obtaining “Ca” in industry	[16,18]
CaO-based Slag	CaO could be reduced to Ca by Si, enhancing P removal	The mechanism of P removal was uncovered	[2,20,21]
Si-Cu alloy coupled with CaO-SiO_2_-CaCl_2_ slag	Impurity P concentrated in both Cu_3_Si phase in the alloy and oxidized to P_2_O_5_ in the slag.	Difficult for P to be oxidized in comparison with impurities of Al, Ca, Mg, and B in MG-Si, and relatively low removal rate of impurity P (32.7–57.6%).	[22]

**Table 2:** Experimental conditions of P removal experiments.

No.	Composition of Alloy (wt.%)			Total Mass of Alloy (g)	Composition of Slag (wt.%)			Total Slag Mass (g)
	Si	Cu	P		CaCl_2_	CaO	CaF_2_	
E0	49	50	1	10	0	0	0	0
E1	49	50	1	10	47.5	5	47.5	10
E2	49	50	1	10	45	10	45	10
E3	49	50	1	10	42.5	15	42.5	10
E4	49	50	1	10	40	20	40	10
E5	49	50	1	10	37.5	25	37.5	10
E6	49	50	1	10	64	20	16	10
E7	49	50	1	10	48	20	32	10
E8	49	50	1	10	32	20	48	10
E9	49	50	1	10	16	20	64	10
E10	59	40	1	10	48	20	32	10
E11	69	30	1	10	48	20	32	10

**Table 3:** EDS quantitative analysis results for Figure 3b.

EDS Analysis	Element (at.%)				Potential Formula
	Si	Cu	Ca	P	
#1	100	-	-	-	Si
#2	24.97	72.98	-	2.05	Cu_3_Si
#3	39.06	35.14	22.77	3.03	CaCu_2_Si_2_
#4	40.67	34.88	21.72	2.74	CaCu_2_Si_2_
#5	20.09	76.68	-	1.23	Cu_3_Si
#6	19.80	74.88	-	0.98	Cu_3_Si
#7	100	-	-	-	Si

- Indicates that the element cannot be detected.

**Table 4:** XRF results of Si-Cu alloy after refining (wt.%).

No.	Si	Cu	P	Ca
E1	47.87	49.45	0.99	1.69
E2	46.87	48.93	0.91	3.29
E3	45.77	48.54	0.87	4.82
E4	44.91	47.95	0.82	6.32
E5	43.81	47.63	0.79	7.77

**Table 5:** Studies on the occurrence state of P in alloys.

Researcher	System	Temperature	Removal Efficiency of P	The State of P in the Alloy
Sun [31]	Si-Al-Ca	T = 1723 K	P: 23→5 ppmw P _removed_ = 78.26%	Dissolved in CaAl_2_Si_2_ phase
Zhu [32]	Si-Al-Ca	T = 1723 K	P:12.3→1.722 ppmw P _removed_ = 86%	Dissolved in CaAl_2_Si_2_ phase
Chen [5]	Si-Al-Sr	T = 1323 K	P: 100→5.34 ppmw P _removed_ = 94.66%	Dissolved in Al_2_Si_2_Sr phase
Huang [18]	Si-Cu-Ca	T = 1823 K	P: 1622→292 ppmw P _removed_ = 82%	Dissolved in CaCu_2_Si_2_ phase

## Data Availability

The original contributions presented in this study are included in the article. Further inquiries can be directed to the corresponding author.
